# Genome-Wide Analysis of the FNSII Gene Family and the Role of CitFNSII-1 in Flavonoid Synthesis in Citrus

**DOI:** 10.3390/plants14131936

**Published:** 2025-06-24

**Authors:** Xinya Liu, Beibei Chen, Ling Luo, Qi Zhong, Chee How Teo, Shengjia Huang

**Affiliations:** 1 Horticulture Research Institute, Sichuan Academy of Agricultural Sciences, Chengdu 610023, China; paul091341@163.com (X.L.); a5363252@163.com (L.L.); zhongqi8707@scsaas.cn (Q.Z.); 2 Key Laboratory of Horticultural Crops Biology and Germplasm Enhancement in Southwest, Ministry of Agriculture and Rural Affairs, Chengdu 611130, China; 3 Key Laboratory for Germplasm Innovation & Utilization of Horticultural Crops of Sichuan Province, Chengdu 610066, China; 4 University Malaya Centre for Research in Biotechnology for Agriculture, Kuala Lumpur 50603, Malaysia; 22050412@siswa.um.edu.my; 5 Institute of Economic Forest Research, Sichuan Academy of Forestry, Chengdu 610081, China

**Keywords:** citrus, hairy roots, CitFNSII-1, flavonoid

## Abstract

Flavonoid synthases (FNSs) are key enzymes catalyzing the conversion of flavanones to flavonoids, yet their functions in citrus remain functionally uncharacterized. In this study, we identified three FNSII genes in the citrus genome. Phylogenetic analysis revealed that citrus FNSII genes share the closest evolutionary distance with apple FNSII genes. Chromosomal localization demonstrated that the three FNSII genes are distributed across two out of nine chromosomes. Gene structure analysis indicated that the majority of motifs within these three FNSII genes are highly conserved. We cloned a gene called *CitFNSII-1* from citrus. Transient overexpression of *CitFNSII-1* in citrus leaves significantly increased flavonoid content, while simultaneous virus-induced silencing of *CitFNSII-1* led to synchronously and significantly reduced gene expression levels and flavonoid content in citrus seedlings. Through the *Agrobacterium rhizogenes*-mediated genetic transformation system, overexpression of *CitFNSII-1* was found to markedly enhance flavonoid accumulation in hairy roots, whereas knockout of *CitFNSII-1* resulted in a significant decrease in flavonoid content in hairy roots. Further experiments verified an interaction between CitFNSII-1 and the Chalcone isomerase-1 (CHI-1) protein. The results demonstrated that the flavonoid accumulation patterns of *CHI-1* and *CitFNSII-1* are highly similar. In conclusion, this study advances the understanding of the flavonoid biosynthesis pathway in citrus and provides a theoretical foundation for molecular breeding strategies in citrus.

## 1. Introduction

Citrus, as one of the most widely cultivated fruits globally, is highly favored by consumers for its unique flavor and rich nutritional components, including carotenoids, vitamin C, folate, dietary fiber, and flavonoids [1]. Functional compounds such as flavonoids are present in citrus fruits, leaves, flowers, and roots [2]. Flavonoids not only influence the color and flavor of citrus fruits but also enhance plant resilience to abiotic and biotic stresses, such as ultraviolet radiation, low temperature, drought, and pathogens [3]. Due to their potent antioxidant properties, flavonoids play significant roles in human health, including anticancer, antiviral, and anti-inflammatory effects [4,5]. Studies have shown that flavonoid accumulation exhibits cultivar specificity; for example, mandarins (*Citrus reticulata*) and sweet oranges (*Citrus sinensis*) accumulate high levels of flavonoids in their peels [6]. As ubiquitous secondary metabolites in plants, extensive in vivo and in vitro studies have confirmed that the unique structures of flavonoids confer biological activity, making them promising candidates for drug development [7]. Consuming more diverse flavonoids can reduce the risk of death and chronic diseases by 6–20%, and the protective effects of intake and diversity on health are independent of each other [8]. Therefore, elucidating the flavonoid biosynthesis pathway in citrus is of great significance for improving the production and application value of these bioactive compounds.

Currently, all identified FNSII proteins in plants almost exclusively belong to the CYP93 subfamily. In monocotyledonous plants, FNSIIs are primarily classified under the CYP93G subfamily, such as CYP93G3 in sorghum (*Sorghum bicolor*) and CYP93G7 in maize (*Zea mays*) [9,10]. In contrast, FNSIIs identified in dicotyledonous plants predominantly belong to the CYP93B subfamily, including CYP93B10 and CYP93B11 in Medicago truncatula, as well as CYP93B16 in soybean (*Glycine max*). Two FNSII genes, *CitFNSII-1* and *CitFNSII-2*, were identified in citrus, belonging to the CYP93B subfamily. These genes catalyze the conversion of flavanones to flavones and are involved in the biosynthesis of polymethoxylated flavones (PMFs) [11]. In Salvia miltiorrhiza, the SmFNSII gene, classified under the CYP93B subfamily, participates in flavonoid biosynthesis [12]. The *Scutellaria baicalensis* genome harbors two FNSII genes (*SbFNSII-1* and *SbFNSII-2*), members of the CYP82D subfamily, which catalyze hydroxylation at the C-6/C-8 positions of flavones, leading to the formation of unique 4′-deoxyflavonoids such as baicalein. In monocots, FNSII enzymes from maize (*Zea mays*) and sorghum (*Sorghum bicolor*) belong to the CYP93G subfamily [13]. These enzymes not only convert flavanones to flavones but also exhibit flavone 2-hydroxylase (F2H) activity, contributing to the synthesis of C-glycosylated flavones [14]. In Arabidopsis thaliana, no typical FNSII genes have been identified; however, members of the CYP93B subfamily may play putative roles in flavonoid metabolism. Research has shown that *SlbHLH95* promotes flavonoid synthesis by directly activating the expression of *SlF3H* and *SlFLS* genes, while inhibiting the expression of the *SlCHS1* gene, thereby regulating flavonoid metabolism [15]. FNSII is an enzyme of the CYP450 family widely present in higher plants, and its identification in citrus species will help improve the synthetic metabolic pathway of citrus flavonoids.

Plant hormones play crucial roles in all stages of plant growth and development. They not only directly regulate plant growth and development but also modulate secondary metabolism, including the biosynthesis of flavonoids. Studies have demonstrated that multiple phytohormones, such as abscisic acid (ABA), gibberellins (GA), methyl jasmonate (MeJA), and methyl salicylate (MeSA), can regulate the synthesis and metabolism of flavonoid compounds [16]. ABA, ethylene, jasmonates, cytokinins, and brassinosteroids promote flavonoid biosynthesis, whereas auxin suppresses this process through negative regulation. Subsequently, transcription factors from the MYB, bHLH, WRKY, NAC, and bZIP families play critical roles in modulating flavonoid biosynthesis [17]. For instance, treatment with MeJA and MeSA has been shown to enhance flavonoid accumulation in *Scutellaria baicalensis* root cultures while simultaneously upregulating the expression of *SbFNSII-2* [18]. Furthermore, MeJA and MeSA have been proven to stimulate flavonoid biosynthesis in tea plants by activating the phenylpropanoid pathway [19]. Previous studies have demonstrated that citrus flavonoids can effectively protect citrus fruits against pathogen attacks. Exogenous SA treatment has been shown to enhance resistance to *Penicillium digitatum* and *Candidatus Liberibacter* asiaticus in citrus fruits, which is associated with the roles of MeSA and MeJA [20]. Therefore, MeSA and MeJA may function by directly modulating flavonoid biosynthesis in citrus, thereby influencing stress resistance responses. However, the specific effects of MeSA and MeJA on citrus flavonoid biosynthesis remain unclear, and whether they regulate the expression of FNSII genes in citrus requires further investigation.

In this study, we conducted the first genome-wide identification and systematic analysis of the FNSII gene family in citrus, encompassing phylogenetic tree construction, gene structure elucidation, chromosomal localization, conserved motif identification, and cis-regulatory element prediction. These findings lay a foundation for elucidating the evolutionary trajectories and biological functions of the citrus FNSII gene family. Through combined validation using transient overexpression, virus-induced gene silencing (VIGS), and *Agrobacterium rhizogenes*-mediated hairy root transformation, we demonstrated that CitFNSII-1 and its interacting protein CHI-1 play critical and functionally similar roles in citrus flavonoid biosynthesis. Furthermore, their expression patterns and regulatory responses to MeSA and MeJA treatments were highly consistent. This study represents the first biological functional validation of citrus FNSII enzymes, significantly advancing our understanding of the flavonoid biosynthetic pathway in citrus.

## 2. Results

### 2.1. Identification and Phylogenetic Tree of FNSII Genes in Citrus

**Figure 1:**
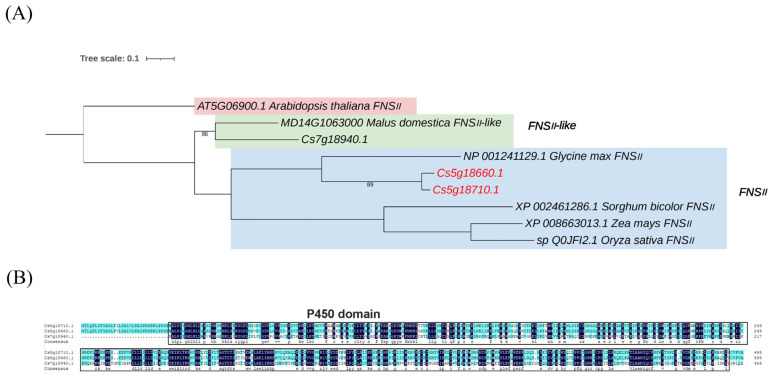
**Identification and analysis of FNSII gene family members in citrus.** (**A**) The phylogenetic tree including FNSII proteins from *Citrus sinensis* and Arabidopsis thaliana. (**B**) Multiple sequence alignment of FNSII proteins in citrus.

Using the HMM profile of the FNSII superfamily as a query, candidate FNSII genes were screened in the citrus genome. A total of three candidate FNSII genes were identified in the citrus genome, and their conserved domains were analyzed via the NCBI database. The results confirmed the presence of three FNSII genes in loquat. A phylogenetic tree of these FNSII gene families from higher plants and citrus was constructed using the maximum likelihood (ML) method, revealing that citrus FNSII genes exhibit the closest evolutionary relationship with apple FNSII genes (Figure 1A).

Multiple sequence alignment of the three FNSII amino acid sequences identified through genome-wide analysis was performed using DNAMAN 9.0 software. The results revealed that the P450 domain (spanning residues 30–493) in all three FNSII proteins contains three characteristic conserved motifs, KESFR, PERF, and PFGTGRRGCPG, confirming their classification within the CYP450 superfamily. Highly conserved amino acid residues, including arginine (R), phenylalanine (F), and glycine (G), were observed within these domains (Figure 1B).

### 2.2. Chromosomal Localization, Synteny Analysis, and Characterizations of the FNSII Genes in Citrus

**Figure 2:**
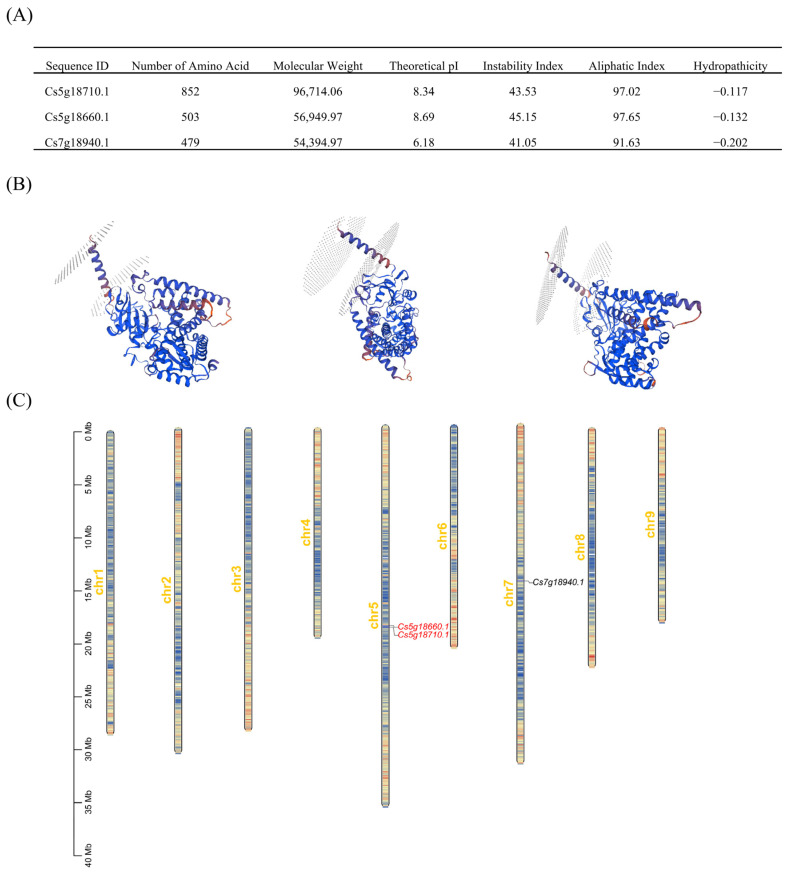
**Chromosome localization, homology analysis, and characterizations analysis of citrus FNSII gene.** (**A**) The characteristics of FNSII genes in citrus. (**B**) Three-dimensional structural diagram of FNSII protein in citrus. (**C**) The chromosomal localization of the FNSII genes.

Physicochemical property analysis revealed that the three FNSII proteins contain 479 to 852 amino acids, with molecular weights ranging from 54,394.97 to 96,714.06 Da. Their isoelectric points (pI) span from 6.18 to 8.34, where *Cs7g18940.1* is an acidic protein, while the others are alkaline. The instability indices ranged from 41.05 to 45.15, classifying all three FNSII proteins as unstable. The aliphatic indices were between 91.63 and 97.65, and the grand average of hydropathicity (GRAVY) values ranged from −0.202 to −0.117 (all < 0), indicating hydrophilic properties (Figure 2A). The corresponding protein three-dimensional structures for *Cs7g18940.1*, *Cs5g18660.1*, and *Cs5g18710.1* were established using the SWISSMODEL website (Figure 2B). Genomic localization analysis showed that two citrus FNSII genes are located on chromosome 5 (chr5). A chromosomal region within 200 kb on chr5 harbors two or more genes, suggesting tandem duplication events. Specifically, *Cs5g18660.1* and *Cs5g18710.1* were identified as tandem duplicates. Additionally, a FNS II-like gene was localized on chromosome 7 (chr7) (Figure 2C).

### 2.3. Conserved Motif and Gene Structure Analysis of the FNSII Genes in Citrus

**Figure 3:**
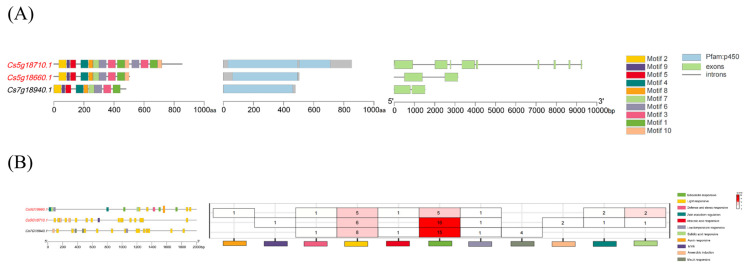
**Conserved motif and gene structure analysis of citrus FNSII gene.** (**A**) Conserved motifs in FNSII proteins are represented by colored boxes. (**B**) UTRs, exons, and introns are represented by green squares, yellow squares, and gray lines, respectively. Black lines indicate length.

Conserved motif analysis revealed that the three FNSII genes exhibit high conservation across most motifs. Notably, with the exception of one FNSII-like gene lacking motif 10, the other two FNSII family members each contain 10 motifs, sharing similar motif distributions but showing slight quantitative variations. The absence of motif 10 in the FNSII-like gene suggests that this motif may play a unique role in specific biological processes mediated by the other two FNSII genes. The loss of motif 10 in the FNSII-like gene might have driven functional divergence or functional constraints, and differences in motif numbers could lead to subtle structural variations between the two FNSII proteins, potentially impacting their biological functions. The domain architectures of the three FNSII genes are largely consistent. Gene structure analysis showed that FNSII genes contain 2–9 exons and 1–8 introns. *Cs5g18710.1* harbors an exceptionally large intron, which may influence its expression, potentially activating or repressing transcription during specific developmental stages (Figure 3A).

All three genes possess a significant number of light-responsive elements. However, *Cs5g18660.1* contains markedly fewer light-responsive elements than the other two genes, suggesting its involvement in light-responsive processes less sensitive to light intensity or photoperiod. *Cs5g18710.1* and *Cs7g18940.1* lack auxin-responsive elements, while *Cs5g18660.1* and *Cs7g18940.1* lack MeJA-responsive elements, indicating divergence in hormonal response regulation among the three genes. *Cs5g18660.1* and *Cs5g18710.1* lack anaerobic induction elements and MYB-binding sites, whereas *Cs7g18940.1* lacks defense- and stress-responsive elements as well as SA-responsive elements, highlighting functional differences in plant defense responses among the three genes (Figure 3B).

### 2.4. Expression of CitFNSII-1 in Citrus

As shown in Supplementary Figure S1, using the expression level of *CitFNSII-1* in a young leaf as a control, the gene expression level in the mature leaf was 3.7 times higher than that in the young leaf (Supplementary Figure S1A). Using the expression level of *CitFNSII-1* in the young fruit stage as a control, the gene expression level in the full ripe stage was upregulated by 3.9 times (Supplementary Figure S1B). Further analysis of the expression characteristics of *CitFNSII-1* in different tissues showed that the expression level of *CitFNSII-1* in peel was 2.8 times higher than that in leaf veins (Supplementary Figure S1C). The expression level of *CitFNSII-1* in *Carrizo citrange* was used as the control, and the gene expression level in *Citrus grandis* was upregulated by 4.7 times (Supplementary Figure S1D).

The results showed that, compared to the control group, exogenous MeSA and MeJA treatments led to a decrease in flavonoid content in citrus leaves (Supplementary Figure S1E,G). Subsequently, qPCR was performed to measure the expression levels of *CitFNSII-1* under MeSA and MeJA treatments to determine whether the reduced flavonoid content was associated with its expression. As shown in Supplementary Figure S1F,H, the transcriptional levels of *CitFNSII-1* in MeSA- and MeJA-treated citrus leaves were significantly downregulated. These results suggested that MeSA and MeJA treatments may reduce flavonoid accumulation by suppressing *CitFNSII-1* expression.

### 2.5. Generation of TRV-CitFNSII-1 Plants

**Figure 4:**
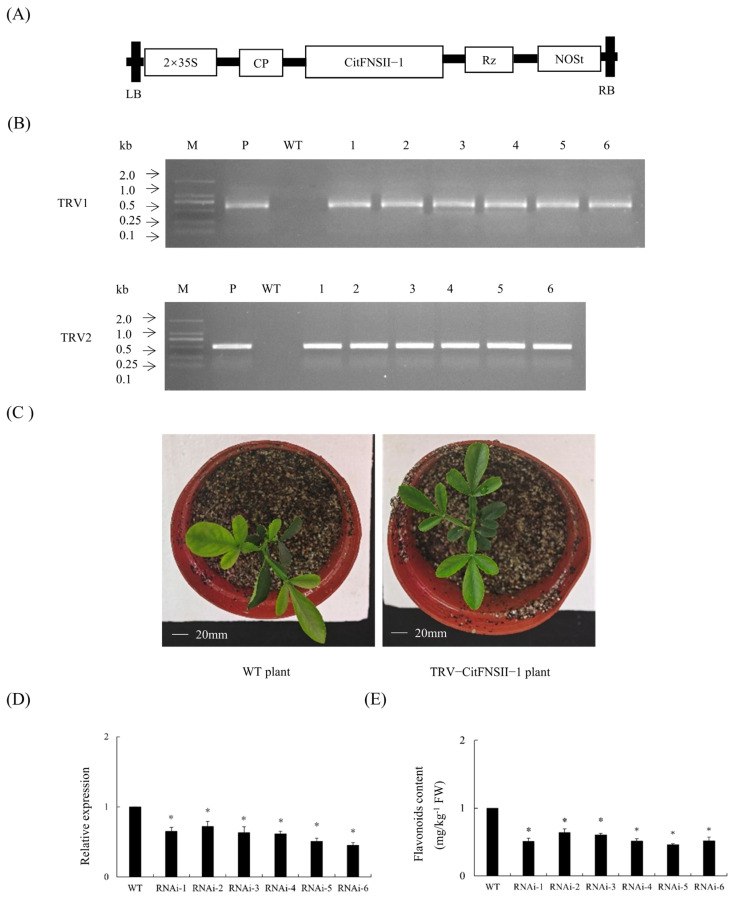
**Transgenic plants silencing *CitFNSII-1*.** (**A**) A 35S, 35S promoter; NOSt, the nopaline synthase terminator; LB, left border; RB, right border. (**B**) Identification of silencing plants by PCR. M, DNA marker; T, TRV plasmid; WT, wild-type control. (**C**) Phenotypic observation of silencing plants. (**D**) Relative expression levels of *CitFNSII-1* in silencing plants. (**E**) Determination of flavonoid content in citrus seedlings with TRV-induced silencing of *CitFNSII-1*. Values are expressed as means ± standard deviation of three independent tests. * on top of the bars indicates significant differences compared to WT control (*p* < 0.05, Student’s *t*-test).

TRV2-*CitFNSII-1* vector was constructed (Figure 4A), and positive plants were identified by PCR, resulting in a total of six TRV2-*CitFNSII-1* plants (Figure 4B). Phenotypic observation revealed that there were no significant differences between TRV2-*CitFNSII-1* plants and WT plants (Figure 4C). The results showed that the gene expression levels of these plants were significantly lower than those of the WT plants (Figure 4D). The significant reduction in *CitFNSII-1* gene expression levels in VIGS plants led to a decrease in flavonoid content (Figure 4E).

### 2.6. Transient Overexpression Analysis of CitFNSII-1 in Citrus Leaves

**Figure 5:**
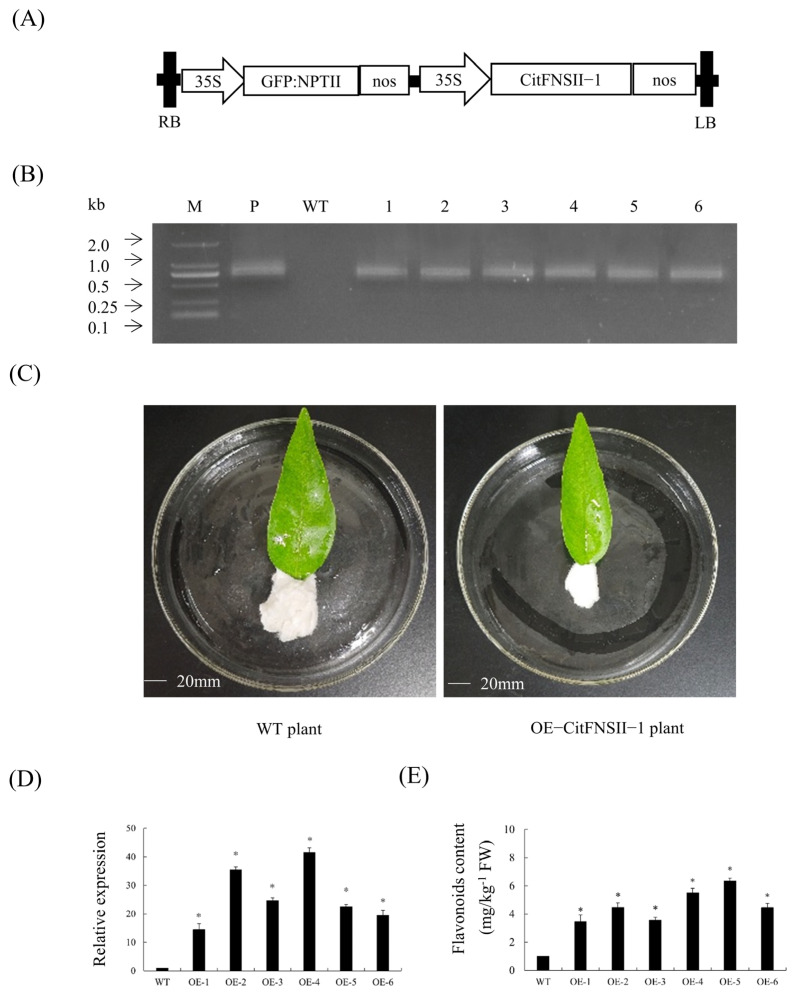
**Gene expression and flavonoid content analysis of transient overexpression of *CitFNSII-1* in citrus leaves.** (**A**) A 35S, 35S promoter; NOSt, the nopaline synthase terminator; LB, left border; RB, right border. (**B**) Identification of transient citrus leaves by PCR. M, DNA marker; P, p35S: *CitFNSII-1* plasmid; WT, wild-type control. (**C**) Observation of symptoms of transient citrus leaves. (**D**) Relative expression levels of *CitFNSII-1* in transient citrus leaves. **(E)** Comparison of flavonoid content in *CitFNSII-1* transiently overexpressing leaves. Values are expressed as means ± standard deviation of three independent tests. * on top of the bars indicates significant differences compared to WT control (*p* < 0.05, Student’s *t*-test).

To rapidly validate the involvement of *CitFNSII-1* in flavonoid biosynthesis in citrus, transient overexpression analysis of *CitFNSII-1* was performed in citrus leaves, and changes in flavonoid content were analyzed. We constructed the *CitFNSII-1* overexpression vector and introduced it into leaves via *Agrobacterium*-mediated vacuum infiltration (Figure 5A). PCR validation confirmed the successful generation of six *CitFNSII-1* transient overexpression leaves (Figure 5B). Phenotypic observation revealed that there were no significant differences between p1300GMN-*CitFNSII-1* plants and WT plants (Figure 5C). The results showed that, compared to the control group injected with the empty vector p1300GMN, leaves infiltrated with *Agrobacterium* carrying p1300GMN-*CitFNSII-1* exhibited significantly elevated relative expression levels of *CitFNSII-1* (Figure 5D) and a marked increase in flavonoid content (Figure 5E). These results provide compelling evidence that *CitFNSII-1* participates in flavonoid biosynthesis in citrus.

### 2.7. Overexpression of CitFNSII-1 in Transgenic Hairy Roots

**Figure 6:**
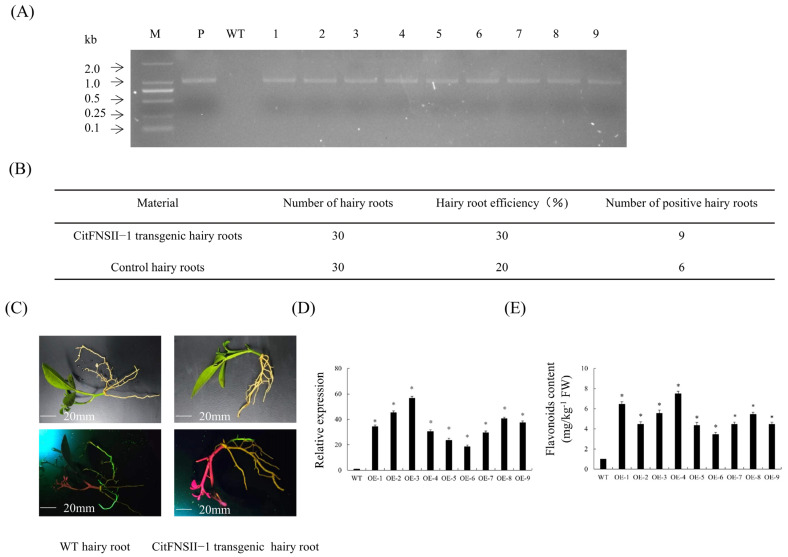
**Transgenic hairy roots overexpressing *CitFNSII-1*.** (**A**) Identification of transgenic hairy roots by PCR. M, DNA marker; P, p35S: *CitFNSII-1* plasmid; WT, wild-type control. (**B**) Statistics of transgenic hairy roots. (**C**) Observation of symptoms of transgenic hairy roots. (**D**) Relative expression levels of *CitFNSII-1* in transgenic hairy roots. (**E**) Comparison of flavonoid content in *CitFNSII-1* overexpressing hairy roots. Values are expressed as means ± standard deviation of three independent tests. * on top of the bars indicates significant differences compared to WT control (*p* < 0.05, Student’s *t*-test).

Nine *CitFNSII-1* transgenic hairy roots were identified by PCR (Figure 6A). The rooting rate of transgenic hairy roots was 30% (Figure 6B). There was no significant difference in phenotype between *CitFNSII-1* transgenic hairy roots and WT hairy roots. *CitFNSII-1* transgenic hairy roots are usually white or yellow, with a length of 7 cm to 12 cm (Figure 6C). The results showed that the gene expression level of *CitFNSII-1* transgenic hairy roots was significantly higher than that of the WT group (Figure 6D). Similarly, the flavonoid content levels in *CitFNSII-1* transgenic hairy roots were significantly higher than those in the WT group (Figure 6E). The results showed that overexpression of *CitFNSII-1* promoted the biosynthesis of flavonoids in citrus hairy roots.

### 2.8. Identification and Editing Efficiency Analysis of CitFNSII-1 in CRISPR/Cas9-Edited Citrus Hairy Roots

**Figure 7:**
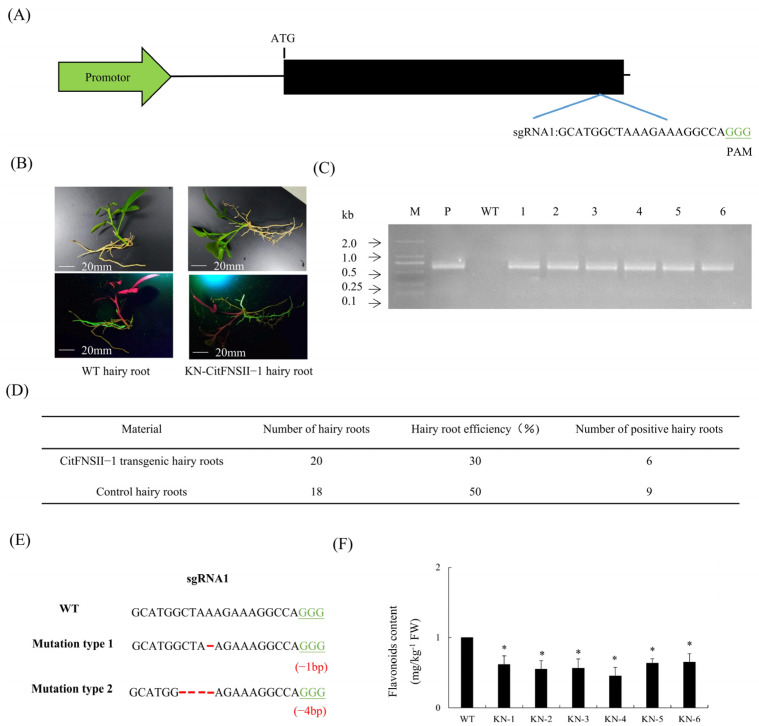
**Identification and phenotypic characterization of gene-edited hairy roots.** (**A**) Schematic diagram of gene editing vector construction. (**B**) Observation of symptoms of transgenic hairy roots. (**C**) Identification of gene-edited hairy roots by PCR. M, DNA marker; P, pKSE401-GFP plasmid; WT, wild-type control. (**D**) Statistics of transgenic hairy roots. (**E**) The green font represents PAM sequences, the red font represents mutated bases, and the red dashed line represents base deletions. (**F**) Comparison of flavonoid content in *CitFNSII-1* gene-edited hairy roots. Values are expressed as means ± standard deviation of three independent tests. * on top of the bars indicates significant differences compared to WT control (*p* < 0.05, Student’s *t*-test).

We selected the target site (sgRNA1: GCATGGCTAAAGAAAGGCCAGGG) to construct the *CitFNSII-1* gene editing vector (Figure 7A). Preliminary phenotypic observation of hairy roots revealed no significant differences in length or size between the gene-edited and wild-type hairy roots (Figure 7B). DNA extracted from green-positive hairy roots was subjected to PCR amplification targeting the Cas9 sequence in the pKSE401-GFP vector, confirming six *CitFNSII-1*-edited hairy roots (Figure 7C) with a positive rate of 30% (Figure 7D). Editing patterns predominantly included one bp insertion and four bp deletions (Figure 7E), with an overall editing efficiency of 100%. Notably, flavonoid content in *CitFNSII-1*-edited hairy roots was significantly lower than in wild-type controls (Figure 7F).

### 2.9. Characteristics of Changes in SA, MeSA, JA, and MeJA Content in CitFNSII-1 Transgenic Hairy Roots

To further investigate the potential regulatory mechanisms of *CitFNSII-1* in hormonal signaling, we compared the hormone content differences between *CitFNSII-1* overexpression and gene-edited hairy roots. The results demonstrated that, in *CitFNSII-1* overexpression hairy roots, the levels of SA, MeSA, JA, and MeJA were significantly downregulated compared to control hairy roots (Supplementary Figure S2A–D). In contrast, in *CitFNSII-1* gene-edited hairy roots, the levels of SA, MeSA, JA, and MeJA were significantly upregulated relative to the control hairy roots (Supplementary Figure S2E–H).

### 2.10. CitFNSII-1 Interacts with CHI-1

**Figure 8:**
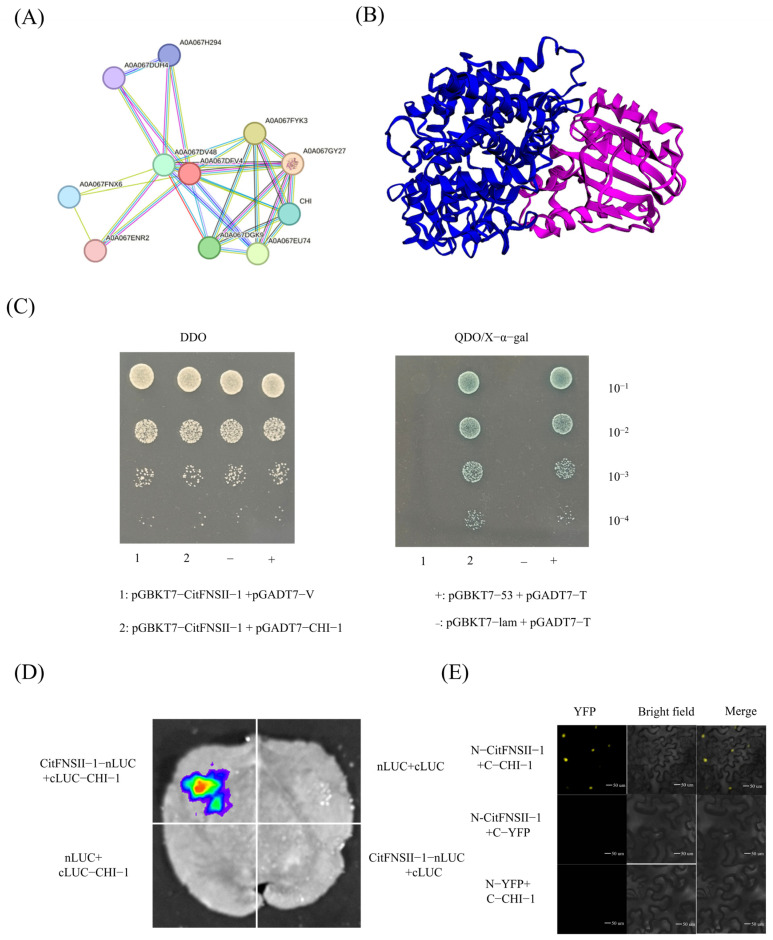
**CitFNSII-1 interacts with CHI-1.** (**A**) Predicting potential interacting proteins of CitFNSII-1 through PPI analysis. (**B**) Using molecular docking technology to predict the complex structure of the interaction between CitFNSII-1 and CHI-1. (**C**) Y2H assay showing that CitFNSII-1 interacts with CHI-1. Positive transformants were spotted onto synthetic defined (SD) medium lacking Trp and Leu and SD medium lacking Trp, Leu, His, and Ade containing x-α-Gal to test protein–protein interaction. (**D**) LCA assay showing that CitFNSII-1 and CHI-1 interact in planta. *N. benthamiana* leaves were co-infiltrated with mixed *Agrobacterium* cultures, each harboring nLUC-CitFNSII-1 or CHI-1-cLUC. (**E**) BiFC assay showing that nYFP-CitFNSII-1 and cYFP-CHI-1 constructs were co-infiltrated in *N. benthamiana* leaves (nYFP-CitFNSII-1 and cYFP-CHI-1 were used as negative controls). Three independent repetitions were carried out with similar results.

Using PPI online prediction of potential interacting proteins of CitFNSII-1 in citrus, it was found that CHI-1 protein may interact with it (Figure 8A). Molecular docking of CitFNSII-1 and CHI-1 was performed using GRAMM software, and the potential complex formed by the two proteins was predicted (Figure 8B). In the Y2H assay, CitFNSII-1 interacted with CHI-1 when CitFNSII-1 was used as the bait (Figure 8C). In the LCA assay, constructs encoding nLUC-CitFNSII-1 and CHI-1-cLUC were infiltrated into *N. benthamiana* leaves, and luminescence signals were observed in the infiltrated regions, confirming their interaction in vivo (Figure 8D). In the BiFC assay, co-expression of YFPn-CitFNSII-1 and YFPc-CHI-1 in *N. benthamiana* leaves resulted in fluorescence signals localized to the nucleus, indicating that these proteins interact within the nucleus (Figure 8E).

### 2.11. Expression of CHI-1 in Citrus

Transcript abundance in mature leaves exhibited a 2.4-fold increase relative to juvenile leaves (Supplementary Figure S3A). During fruit development, *CHI-1* expression demonstrated a 2.8-fold elevation at full ripeness compared to the young fruit stage (Supplementary Figure S3B). Expression analysis identified preferential accumulation in peel tissue, with a 3.5-fold higher level than leaf veins (Supplementary Figure S3C). The results showed that *Citrus grandis* displayed 3.3-fold enhanced *CHI-1* mRNA levels compared to *Carrizo citrange* (Supplementary Figure S3D).

The results demonstrated that exogenous MeSA and MeJA treatments induced a concomitant reduction in leaf flavonoid content (Supplementary Figure S3E,G). Transcriptional analysis via RT-qPCR revealed significant downregulation of *CHI-1* under both treatments (Supplementary Figure S3F,H). The results suggested that MeSA/MeJA-mediated suppression of *CHI-1* expression contributes to compromised flavonoid biosynthesis.

### 2.12. Generation of TRV-CHI-1 Plants

**Figure 9:**
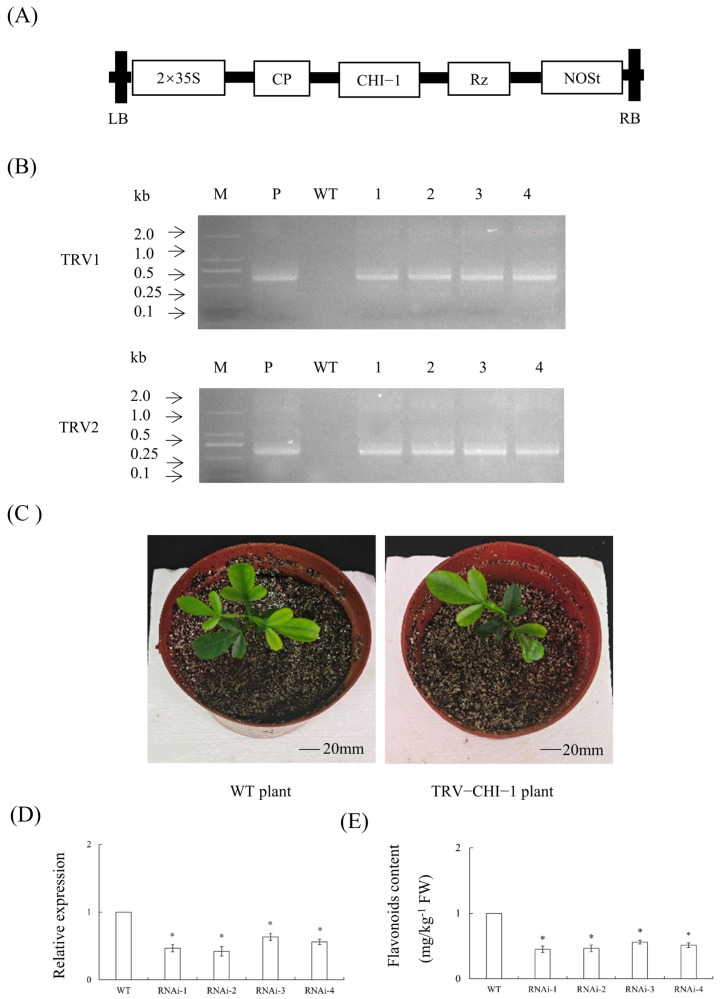
**Transgenic plants silencing *CHI-1*.** (**A**) A 35S, 35S promoter; NOSt, the nopaline synthase terminator; LB, left border; RB, right border. (**B**) Identification of silencing plants by PCR. M, DNA marker; T, TRV plasmid; WT, wild-type control. (**C**) Phenotypic observation of silencing plants. (**D**) Relative expression levels of *CHI-1* in silencing plants. (**E**) Determination of flavonoid content in citrus seedlings with TRV-induced silencing of *CHI-1*. Values are expressed as means ± standard deviation of three independent tests. * on top of the bars indicates significant differences compared to WT control (*p* < 0.05, Student’s *t*-test).

The TRV2-*CHI-1* vector was successfully generated (Figure 9A), with four transgenic plants confirmed through PCR (Figure 9B). Phenotypic observation revealed that there were no significant differences between TRV2-*CHI-1* plants and WT plants (Figure 9C). The results showed that the gene expression levels of these plants were significantly lower than those of the WT plants (Figure 9D). The significant reduction in *CHI-1* gene expression levels in VIGS plants led to a decrease in flavonoid content (Figure 9E).

### 2.13. Transient Overexpression Analysis of CHI-1 in Citrus Leaves

**Figure 10:**
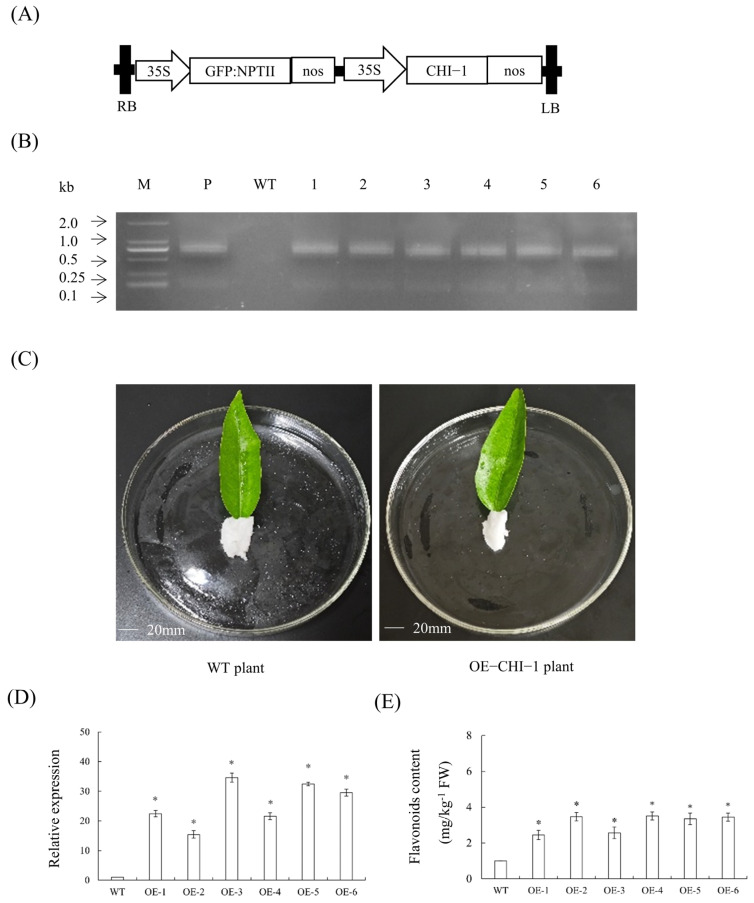
**Gene expression and flavonoid content analysis of transient overexpression of *CHI-1* in citrus leaves.** (**A**) A 35S, 35S promoter; NOSt, the nopaline synthase terminator; LB, left border; RB, right border. (**B**) Identification of transient citrus leaves by PCR. M, DNA marker; P, p35S: *CHI-1* plasmid; WT, wild-type control. (**C**) Observation of symptoms of transient citrus leaves. (**D**) Relative expression levels of *CHI-1* in transient citrus leaves. (**E**) Comparison of flavonoid content in *CHI-1* transiently overexpressing leaves. Values are expressed as means ± standard deviation of three independent tests. * on top of the bars indicates significant differences compared to WT control (*p* < 0.05, Student’s *t*-test).

To functionally investigate CHI-1’s role in citrus flavonoid metabolism, we conducted transient expression experiments in foliar tissues. The *CHI-1* coding sequence was cloned into a plant expression vector (Figure 10A) and delivered into leaf tissues through *Agrobacterium*-mediated vacuum infiltration. PCR-based screening generated six overexpressing plants (Figure 10B). Phenotypic observation revealed that there were no significant differences between p1300GMN-*CHI-1* plants and WT plants (Figure 10C). The results showed that leaves expressing the p1300GMN-*CHI-1* construct demonstrated substantially enhanced *CHI-1* transcription levels relative to empty vector-transfected controls (Figure 10D). This transcriptional upregulation corresponded with measurable increases in flavonoid accumulation (Figure 10E). The results indicated CHI-1’s functional participation in the flavonoid biosynthetic pathway of citrus plants.

### 2.14. Overexpression of CHI-1 in Transgenic Hairy Roots

**Figure 11:**
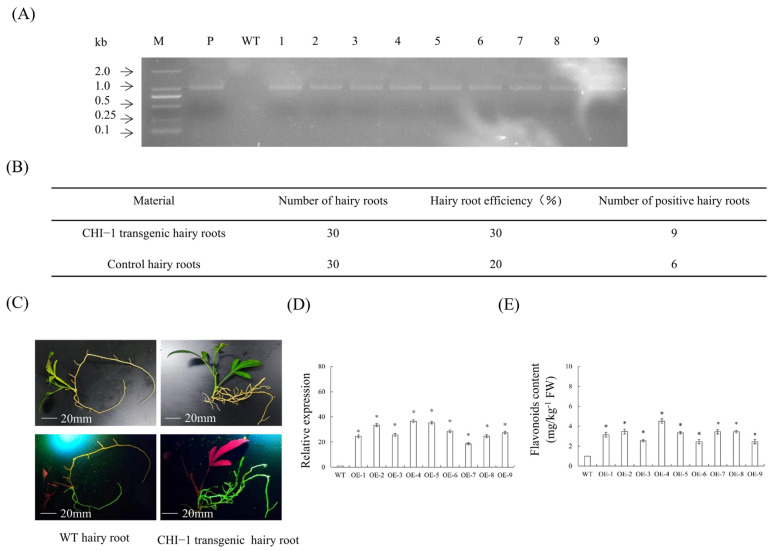
**Transgenic hairy roots overexpressing *CHI-1*.** (**A**) Identification of transgenic hairy roots by PCR. M, DNA marker; P, p35S: CHI-1 plasmid; WT, wild-type control. (**B**) Statistics of transgenic hairy roots. (**C**) Observation of symptoms of transgenic hairy roots. (**D**) Relative expression levels of *CHI-1* in transgenic hairy roots. (**E**) Comparison of flavonoid content in *CHI-1* overexpressing hairy roots. Values are expressed as means ± standard deviation of three independent tests. * on top of the bars indicates significant differences compared to WT control (*p* < 0.05, Student’s *t*-test).

PCR screening successfully identified nine *CHI-1* transgenic hairy roots (Figure 11A), exhibiting a 30% root induction efficiency (Figure 11B). Morphological comparison revealed no observable phenotypic variations between transgenic and WT hairy roots. Both transgenic and WT hairy roots displayed similar coloration (white to yellow) and size characteristics, ranging from 5 to 14 cm in length (Figure 11C). The results showed markedly enhanced *CHI-1* transcript accumulation in transgenic hairy roots relative to WT controls (Figure 11D). Consistent with gene expression patterns, transgenic roots showed corresponding increases in flavonoid content compared to WT controls (Figure 11E). The results indicated that *CHI-1* overexpression enhances flavonoid biosynthesis in citrus hairy roots.

### 2.15. Identification and Editing Efficiency Analysis of CHI-1 in CRISPR/Cas9-Edited Citrus Hairy Roots

**Figure 12:**
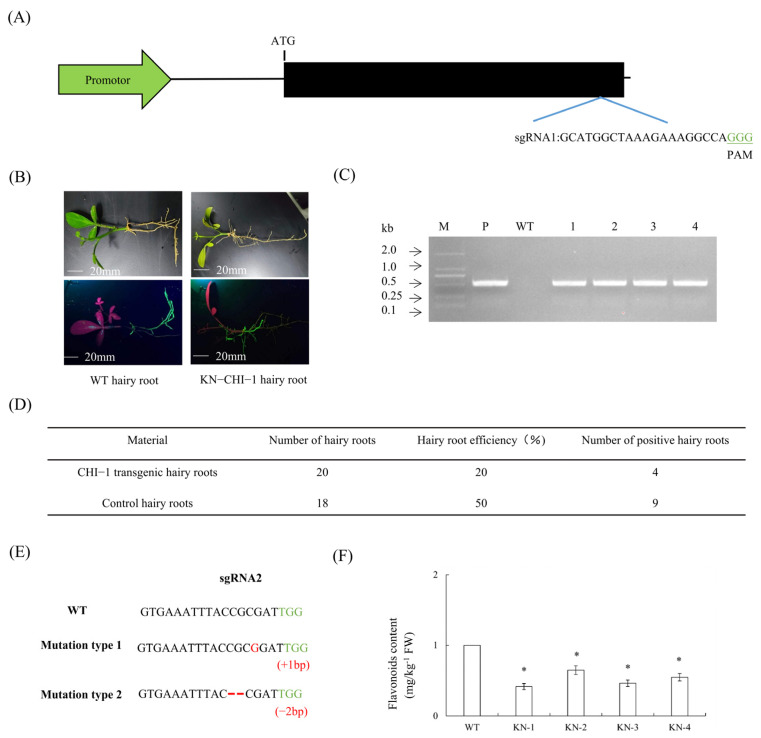
**Identification and phenotypic characterization of gene-edited hairy roots.** (**A**) Schematic diagram of gene editing vector construction. (**B**) Observation of symptoms of transgenic hairy roots. (**C**) Identification of gene-edited hairy roots by PCR. M, DNA marker; P, pKSE401-GFP plasmid; WT, wild-type control. (**D**) Statistics of transgenic hairy roots. (**E**) The green font represents PAM sequences, the red font represents mutated bases, and the red dashed line represents base deletions. (**F**) Comparison of flavonoid content in *CHI-1* gene-edited hairy roots. Values are expressed as means ± standard deviation of three independent tests. * on top of the bars indicates significant differences compared to WT control (*p* < 0.05, Student’s *t*-test).

A CRISPR/Cas9-based editing construct targeting *CHI-1* was engineered using the designed sgRNA2 (GTGAAATTTACCGCGATTGG) (Figure 12A). Preliminary phenotypic observation of hairy roots revealed conserved growth characteristics between edited and WT hairy roots, with comparable length and diameter parameters (Figure 12B). PCR-based screening using Cas9-specific primers confirmed four edited hairy roots derived from GFP-positive root tissues (Figure 12C), representing a 30% transformation efficiency (Figure 12D). Editing patterns predominantly including one bp insertion and two bp deletions (Figure 12E). The results showed reduced flavonoid accumulation in edited hairy roots relative to WT hairy roots (Figure 12F).

### 2.16. Characteristics of Changes in SA, MeSA, JA, and MeJA Content in CHI-1 Transgenic Hairy Roots

To elucidate CHI-1’s regulatory interactions with phytohormonal pathways, we conducted phytohormone profiling in *CHI-1* overexpression and CRISPR-edited hairy roots. The results revealed coordinated downregulation of SA, MeSA, JA, and MeJA in overexpression hairy roots compared to WT controls (Supplementary Figure S4A–D). Conversely, CRISPR-mediated *CHI-1* suppression resulted in increased levels of these hormones relative to WT hairy roots (Supplementary Figure S4E–H).

## 3. Discussion

To investigate the functional architecture and regulatory mechanisms of the citrus FNSII gene, this study first performed genome-wide bioinformatics analysis of the FNSII gene family in citrus, followed by functional validation of the target gene *CitFNSII-1*. Transient overexpression and VIGS techniques were employed alongside *Agrobacterium rhizogenes*-mediated genetic transformation to generate *CitFNSII-1* transgenic materials. Quantitative analysis of physiological parameters and flavonoid content in transgenic plants was conducted to elucidate the regulatory role of *CitFNSII-1* in citrus flavonoid biosynthesis.

Functional FNSII genes have been identified across diverse plant systems, including cereal crops, medicinal plants, ornamental plants, and fruit trees [21,22,23]. All currently characterized plant FNSII proteins belong to the CYP93 subfamily, which also includes flavonoid 2-hydroxylases (F2Hs). These F2Hs catalyze the hydroxylation of flavanones at the C2 position to generate 2-OH flavanones, serving as precursors for flavonoid-C-glycoside biosynthesis [24,25]. Furthermore, FNSII has been demonstrated to function as an essential component of flavonoid metabolic complexes, enhancing flavonoid biosynthesis through biochemical coordination [26]. As a CYP450 family enzyme ubiquitously present in higher plants, the characterization of FNSII in citrus species will contribute to the systematic elucidation of flavonoid biosynthetic pathways in Rutaceae. However, genome-wide identification and functional characterization of FNSII genes remain unreported in citrus. This study completed the whole genome identification of the citrus FNSII gene family for the first time and explored the mechanism of *CitFNSII-1* in citrus flavonoid synthesis.

Phylogenetic analysis revealed a high conservation of *CitFNSII-1* during evolutionary processes, suggesting potential gene duplication events. Previous studies demonstrated a dynamic accumulation pattern of flavonoids in citrus fruit development, characterized by an initial increase followed by a gradual decline [27]. Comparative analysis across cultivars showed the highest flavonoid content in mandarin (*Citrus reticulata*) peels, intermediate levels in sweet orange (*C. sinensis*), and negligible accumulation in pomelo (*C. grandis*). Notably, Satsuma mandarin exhibited significantly elevated flavanone content compared with Ponkan mandarin *(Citrus reticulata ‘Ponkan’*) [28]. Further investigation identified *CitFNSII-1* as a duplicated gene whose expression strongly correlated with interspecific flavonoid variation and showed stage-specific associations with flavonoid accumulation during fruit and leaf development.

Flavonoids modulate hormonal signaling pathways, including those involving IAA [29], ABA [30], and SA [31] signaling pathways. The phytohormone MeSA has been documented to regulate flavonoid metabolism [32]. In this study, MeSA and MeJA treatments resulted in significantly reduced flavonoid accumulation. Further analysis revealed concomitant downregulation of *CitFNSII-1* expression, indicating heightened sensitivity of *CitFNSII-1* to both MeSA and MeJA, potentially through similar regulatory mechanisms. The results indicated that flavonoid depletion directly correlates with *CitFNSII-1* suppression, confirming its central role in flavonoid biosynthesis. Analogously, MeJA specifically induces *SbFNSII-2* expression to promote root-specific flavonoid synthesis in *Scutellaria baicalensis*, while MeSA and MeJA likely activate convergent metabolic pathways in *Bidens pilosa* leaves [33]. In tea (*Camellia sinensis*), 1 mmol/L MeSA enhances flavonoid production via phenylpropanoid pathway activation [32], paralleled by SA-induced upregulation of flavonoid biosynthetic genes (F3H, DFR) and increased flavonoid content in wheat (*Triticum aestivum*) leaves [34], aligning with our experimental results. Nevertheless, the precise molecular mechanisms underlying MeSA/MeJA-mediated flavonoid reduction in citrus require further investigation.

Methyl salicylate (MeSA), a volatile derivative of salicylic acid (SA), acts as a key signaling molecule for systemic acquired resistance (SAR) in citrus. However, MeSA concurrently suppresses the biosynthesis of secondary metabolites (including flavonoids) to reallocate metabolic resources toward combating pathogens or abiotic stressors [35]. Experimental evidence demonstrates that MeSA treatment significantly reduces total flavonoid content in citrus and downregulates *CitFNSII-1* expression. This inhibition likely stems from preferential energy allocation to non-flavonoid protective metabolites. MeJA strongly induces lignin monomer biosynthesis genes (e.g., Phenylalanine ammonia-lyase (PAL) and Cinnamoyl-CoA reductase (CCR)), thereby depleting shared precursors like coumaroyl-CoA. Consequently, flavonoid synthase activity (e.g., FNSII and CHI) decreases due to substrate limitations. Additionally, MeJA treatment activates antioxidant enzymes (e.g., Superoxide dismutase (SOD) and Catalase (CAT)), enhancing endogenous antioxidant capacity. This reduces plant reliance on flavonoids (exogenous antioxidants), leading to feedback inhibition of their synthesis [36].

Flavonoids, as a vital class of secondary metabolites, are ubiquitously present in terrestrial plants. CHI, a key rate-limiting enzyme, catalyzes the stereospecific isomerization of chalcones into corresponding flavanones [37]. CHI typically exists as a multigene family and is classified into four functional types (I-IV) based on biochemical activity [38]. Since the first identification of CHI from Phaseolus vulgaris cell cultures [39], CHI genes in higher plants, including *Arabidopsis thaliana* [40], *Lotus japonicus* [41], and *Solanum lycopersicum* [42], have been systematically cloned and functionally characterized, demonstrating their role in promoting flavonoid biosynthesis.

Transcription factors including MYB, bHLH, and AP2/ERF regulate CHI expression by binding to its promoter. For instance, AP2/ERF family members enhance flavonoid accumulation through transcriptional activation of CHI genes [43]. In leguminous plants, CHI-1 interacts with symbiotic-related transcription factors (NSP2) to drive isoflavone biosynthesis, facilitating rhizobial recruitment [44]. Environmental stressors such as low temperature and UV-B radiation upregulate CHI expression to promote flavonoid synthesis. For example, cold stress induces the interaction between apple MdMYB308L and CHI, enhancing anthocyanin accumulation to mitigate chilling injury [45]. CHI competes with FNS for flavanone substrates, thereby directing metabolic flux toward either flavone or anthocyanin biosynthesis pathways [46]. Heterologous overexpression of CHI significantly increases flavonoid content, as demonstrated by a threefold elevation in pericarp flavones observed in tomato expressing petunia CHI [47]. These results collectively validate CHI’s role in promoting flavonoid biosynthesis, consistent with the biological function of citrus CHI genes elucidated in our experiment.

This study focused on changes in total flavonoid content but did not resolve specific alterations in flavonoid subtypes. PMFs constitute key bioactive components in citrus peels, yet whether *CitFNSII-1*’s catalytic efficiency toward different substrates affects PMF biosynthesis remains unclear. The lack of targeted metabolomics analysis (LC-MS/MS) precluded definitive determination of *CitFNSII-1*’s functional specialization in synthesizing specific flavonoid subclasses [48]. Experiments utilized only a limited number of citrus varieties (primarily leaves and hairy roots), failing to cover high-flavonoid cultivars or critical fruit developmental stages. Given significant inter-varietal differences in flavonoid accumulation patterns, the generalizability of conclusions requires further validation [49]. We combined targeted metabolomics with single-cell transcriptomics to map *CitFNSII-1* expression profiles and flavonoid subtype distribution in specialized tissues (oil glands in fruit peel, leaf veins). We incorporated high-flavonoid germplasms (Satsuma mandarin) and low-accumulation germplasms (pummelo) to analyze correlations between CitFNSIIs allelic variations and flavonoid phenotypes [50]. In summary, while this work reveals *CitFNSII-1*’s central role in citrus flavonoid biosynthesis, the complexity of its metabolic network (subtype differentiation and environmental interactions) requires systematic resolution. Future studies must adopt integrated strategies spanning diverse cultivars, multi-omics approaches, and gene editing to precisely identify breeding targets and establish an end-to-end theoretical framework for developing high-flavonoid citrus varieties.

## 4. Materials and Methods

### 4.1. Plant Materials, Microbial Strains, and Growth Conditions

The experimental materials such as citrus (*Poncirus trifoliata* × *Citrus sinensis* (citrange)) and *Nicotiana benthamiana* were taken from the greenhouse of the Horticulture Research Institute at the Sichuan Academy of Agricultural Sciences. Citrus seedlings subjected to vacuum infiltration treatment, along with leaves and hairy roots, were cultivated in vermiculite alongside *Nicotiana benthamiana* in a controlled-environment growth chamber maintained at 22 °C under long-day conditions (16 h light/8 h dark photoperiod). The *Escherichia coli* strain DH5α was cultured in Luria–Bertani (LB) medium at 37 °C, while *Agrobacterium* strains EHA105, K599, and GV3101 were grown in LB medium supplemented with 50 µg/mL kanamycin at 28 °C.

### 4.2. Identification of the FNSII Genes in Citrus

The Hidden Markov Model (HMM) of the Homeobox (HOX) superfamily (PF00046) was obtained from the Pfam database (http://pfam.xfam.org/). The FNSII protein sequences of the model plant *Arabidopsis thaliana* were retrieved from the TAIR database (https://www.arabidopsis.org/). Protein sequences of the FNSII gene family from previously reported higher plants were acquired from the NCBI database (https://www.ncbi.nlm.nih.gov/). Candidate FNSII gene family sequences were identified through bidirectional BLAST alignment with published citrus genome sequences. The presence of the conserved P450 domain was verified using the NCBI Conserved Domain Database (CDD) (https://www.ncbi.nlm.nih.gov/cdd/).

### 4.3. Phylogenetic Tree, Multiple Sequence Alignment, and Characterizations Analysis of the FNSII Proteins

Multiple sequence alignment of the amino acid sequences of all FNSII family members was conducted using MEGA 7.0 [51]. The aligned sequences were trimmed with trimAI, and a phylogenetic tree was constructed using the maximum likelihood (ML) method in IQ-TREE. The resulting tree was visualized and annotated using the ITOL online platform (https://itol.embl.de). Multiple sequence alignment of citrus FNSII proteins was further analyzed using DNAMAN software (v9.0) with default parameters [52]. Protein characteristics, including coding sequence length, theoretical isoelectric point (pI), molecular weight, and amino acid length, were predicted using the ExPASY database (https://web.expasy.org/protparam/) [53].

### 4.4. Chromosomal Localization, Gene Structure, Conserved Motif, and Synteny Analysis of the FNSII Genes in Citrus

The citrus genome files were downloaded from the NCBI database, and the genome annotation file (GFF format) was obtained. Using TBtools-II, the annotation files of citrus FNSII gene family members were extracted, and chromosomal localization visualization was performed. The intron–exon structures of citrus FNSII genes were analyzed based on genomic sequences and coding sequences. Conserved protein motifs were identified using the MEME suite (https://meme-suite.org/meme/tools/meme) [54]. Structural domains of citrus FNSII proteins were predicted via the NCBI CDD (https://www.ncbi.nlm.nih.gov/Structure/bwrpsb/bwrpsb.cgi). Finally, gene structures, domains, and conserved motifs of citrus FNSII genes were visualized using TBtools-II [55].

### 4.5. Cloning and Sequence Analysis of Genes and Promoter

The 2000 bp upstream sequences of citrus FNSII genes were extracted using TBtools-II software. Promoter prediction analysis was performed via the PlantCARE online platform (https://bioinformatics.psb.ugent.be/webtools/plantcare/html/). Subsequently, the distribution and abundance of cis-acting elements were visualized using TBtools and the ggplot2 package [56].

### 4.6. RT-qPCR Analysis

Total RNA was extracted using the EASYspin Plus Plant RNA Extraction Kit (Aidlab, Shanghai, China). cDNA synthesis was performed with the PrimeScript™ RT Reagent Kit with gDNA Eraser (Takara Bio, Dalian, China). qPCR was carried out using NovoStart^®^ SYBR qPCR SuperMix Plus (Novoprotein, Shanghai, China). qPCR primers for *CitFNSII-1* and *CHI-1* genes were designed using Primer Blast in NCBI (Supplementary Table S1). Using a young leaf, the young fruit stage, a vein, and *carrizo citrange* as references, the relative expression level of *CitFNSII-1* and *CHI-1* genes was calculated using the 2^−ΔΔCt^ method. The test was repeated three times.

### 4.7. Exogenous MeSA and MeJA Treatment of Citrus Leaves and Fruit

Uniform-sized citrus leaves and fruits free of mechanical damage were selected. A 5 mL volume of 1 mM MeSA and MeJA was injected into one side of each sample as the treatment group, while an equal volume of distilled water was injected into the opposite side as the control group [57]. Each treated fruit or leaf constituted one biological replicate, with five biological replicates established. Treated samples were stored at room temperature for one week. The injected regions were subsequently excised, cut into small pieces, snap-frozen in liquid nitrogen, and stored at −80 °C for subsequent flavonoid content quantification and gene expression analysis. The test was repeated three times.

### 4.8. Vectors Construction

TRV2*-CitFNSII-1* and TRV2*-CHI-1* primers were designed (Supplementary Table S1), and PCR amplification was performed using pGEM-Teasy containing *CitFNSII-1* and *CsCHI-1* interfering fragments as templates. P1300GMN*-CitFNSII-1* and P1300GMN*-CHI-1* primers were designed (Supplementary Table S1), and pGEM-Teasy plasmid containing the CDS sequence of *CitFNSII-1* and *CHI-1* was used as a template for PCR amplification. The recombinant plasmid was transformed into *E. coli* DH5α, and positive clones were screened by sequencing. For *CitFNSII-1* and *CHI-1*-CRISPR, one sgRNA targeting the exon of *CitFNSII-1* (sgRNA1: CCATACGAGCAGTTACGAAG) and one sgRNA targeting the exon of *CHI-1* (sgRNA2: TGTTGAGCCAGCCAAAGGAC) were designed using the web server CRISPR-P [58] and cloned into the binary vector pKSE401G [59] by golden gate assembly.

### 4.9. Citrus Transformation

The TRV-mediated VIGS experiment in citrus was conducted following a previously established method [60]. *Agrobacterium tumefaciens* cultures carrying the TRV1 with TRV2*-CitFNSII-1* and TRV2*-CHI-1* constructs were grown in liquid LB medium to an optical density (OD600) of 0.8, followed by centrifugation and resuspension in an infiltration buffer containing 10 mmol/L MES, 10 mmol/L MgCl_2_, and 200 μmol/L acetosyringone (AS). Citrus seedlings were vacuum-infiltrated with a 1:1 mixture of *Agrobacterium* cultures harboring TRV1 with TRV2*-CitFNSII-1* and TRV2*-CHI-1* constructs, while seedlings infiltrated with TRV1 and an empty TRV2 vector served as the control. After infiltration, the seedlings were cultivated in darkness for 3 days and then transferred to a photoperiod of 16 h light/8 h dark until root establishment, followed by an additional month of growth in a greenhouse.

The transient overexpression experiment was conducted following a previously described method [61]. Transformed strains carrying recombinant vectors and the empty vector were first cultured in liquid LB medium at 28 °C, followed by centrifugation and resuspension according to the protocol outlined in the VIGS experiment. Bacterial suspensions containing the target genes and the control were infiltrated into citrus leaves. The suspensions were resuspended in a buffer and infiltrated into the leaves. After infiltration, the leaves were cultivated in darkness for 24 h and then subjected to a 16 h light/8 h dark photoperiod for 3 days.

*Agrobacterium rhizogenes*-mediated hairy root transformation assay was referenced from a previously established method [62]. Citrus branches with diameters of approximately 0.5 cm were collected and cut into stem segments (~5 cm in length) containing one or more axillary buds using a sterilized blade. Root systems were removed, and hypocotyls were retained. Bacterial suspensions (OD_600_ = 0.6–0.8) were infiltrated into hypocotyl incisions via vacuum infiltration for 30 min. The hypocotyls were then inserted into moist vermiculite and cultivated in a constant temperature incubator at 22 °C under a 16 h light/8 h dark photoperiod.

### 4.10. Determination of Hormone Content

Three hairy roots from each transgenic line were collected, ground into a fine powder in liquid nitrogen, and homogenized. The contents of SA, MeSA, JA, and MeJA in the supernatant were quantified using plant enzyme-linked immunosorbent assay (ELISA) kits (Jiweibio, Shanghai, China). Absorbance (OD) at 450 nm was measured using a SpectraMax^®^ M2 microplate reader (Molecular Devices Corporation, Menlo Park, CA, USA) [63]. Hormone concentrations per gram of hairy root fresh weight were calculated using Excel 365. All analyses ensure three biological and technical replicates.

### 4.11. Flavonoids Extraction and Measurement

The extraction and quantification of flavonoids were conducted according to the previously described method, with three biological and technical replicates. Briefly, 0.2 g of leaf or hairy root tissue was ground into a fine powder in liquid nitrogen and transferred to a centrifuge tube. A 700 μL aliquot of extraction solvent (methanol: DMSO = 1:1, *v*/*v*) was added, and the mixture was vortexed thoroughly, followed by ultrasonic extraction for 30 min. After centrifugation at 12,000 rpm for 10 min, the supernatant was collected. The pellet was re-extracted with 700 μL of the same solvent twice, and all supernatants were pooled. The combined supernatant was adjusted to a final volume of 2.5 mL with methanol, filtered through a 0.22 μm membrane, and stored in the dark for subsequent analysis.

Flavonoid separation was performed using a high-performance liquid chromatography (HPLC) system equipped with an Xbridge UPLCC18 column (5 μm particle size, 4.6 × 150 mm). Mobile phase A consisted of water containing 0.2% acetic acid, and mobile phase B was methanol. A gradient elution program was applied with an injection volume of 5 μL, column temperature of 24 °C, and flow rate of 0.8 mL/min. Detection wavelengths were set at 283 nm and 330 nm. The test was repeated three times.

### 4.12. Identification of Positive Transgenic Plants and Detection of Editing Efficiency

Genomic DNA was extracted from putative transgenic plants. Positive transgenic plants were confirmed at the DNA level by amplifying the sequence of the pKSE401 vector using specific primers (Supplementary Table S1). To assess editing efficiency, the target gene regions were PCR-amplified and subjected to Sanger sequencing to analyze editing patterns. The PCR products were subsequently cloned into the pTOPO-T vector, and single colonies were selected for sequencing to calculate editing efficiency.

### 4.13. Homology Modeling and Molecular Docking

PPI (https://cn.string-db.org/cgi/network) online prediction of potential interacting proteins of CitFNSII-1 in citrus was used. To establish suitable protein templates for homology modeling, the amino acid sequences of CitFNSII-1 and CHI-1 were individually queried on the SWISS-MODEL platform (https://swissmodel.expasy.org/). Three-dimensional (3D) homology models of CitFNSII-1 and CHI-1 were generated using the SWISS-MODEL server. Molecular docking was performed following a previous report [64] with the GRAMM software (https://gramm.compbio.ku.edu/request) [65].

### 4.14. Yeast Two-Hybrid (Y2H) Assay

Using the pGEM-T-*CitFNSII-1* plasmid as a template, the *CitFNSII-1* gene was amplified using primer BD-*CitFNSII*-1-F/R (Supplementary Table S1). *CitFNSII-1* was ligated to a pGBKT7 bait vector using the homologous recombination method and transformed into *Escherichia coli* DH5α, positive clones were identified through PCR amplification and sequencing, and the pGBKT7-*CitFNSII-*1 plasmid was constructed. The construction of the pGADT7-*CHI*-1 prey vector is the same as above.

The pGADT7-*CHI*-1 vector was co-transformed with the pGBKT7-*CitFNSII*-1 plasmid into Y2Hgold yeast. pGBKT7-53 was co-transformed with pGADT7-T, pGBKT7-Lam, and pGADT7-T as positive and negative controls, respectively. The bacterial solution was then coated on DDO/X (SD/-Leu/-Trp), TDO (SD/-Leu/-Trp/-His), and QDO/X (SD/-Leu/-Trp/-His/Ade /X-α-gal) media. The test was repeated three times.

### 4.15. Bimolecular Fluorescence Complementation Assay

The coding sequence of *CitFNSII-1* was cloned into the n-YFP vector, and the coding sequence of *CHI-1* was cloned into the c-YFP vector. Primers used for cloning are listed in Supplementary Table S1. The constructed fusion vectors or empty vectors were transformed into *Agrobacterium tumefaciens* strain GV3101. All vectors were verified by Sanger sequencing prior to transformation into *A. tumefaciens* GV3101. Bacterial suspensions (mixed at a 1:1 *v*/*v* ratio and OD = 0.6–0.8) were co-infiltrated into 4-week-old *Nicotiana benthamiana* leaves. Transiently expressed fusion proteins were observed via confocal laser scanning microscopy 72 h post-infiltration. Three independent biological replicates were performed.

### 4.16. Luciferase Complementation Assay

The amplified coding sequence (CDS) of *CitFNSII-1* (without the stop codon) was ligated into the nLuc vector to construct the fusion protein *CitFNSII-1*-nLUC. The CDS of *CHI-1* was ligated into the cLUC vector to construct the fusion protein *CHI*-1-cLUC. Recombinant plasmids were transformed into *Agrobacterium tumefaciens* strain GV3101. Agrobacterium suspensions carrying the respective constructs were mixed at a 1:1 ratio and co-infiltrated into *Nicotiana benthamiana* leaves [66]. Luminescence signals resulting from reconstituted luciferase activity were detected using the IndiGo™ in vivo molecular imaging system 48 h post-infiltration. Three independent biological replicates were performed.

### 4.17. Statistics Analysis

All experiments were performed with a minimum of three independent biological replicates, and data were expressed as mean ± standard error (SE). Statistical analyses included one-way analysis of variance (ANOVA) followed by Duncan’s multiple-range test for multi-group comparisons, while pairwise comparisons were assessed using Student’s *t*-test. Statistical significance was evaluated with SPSS version 26 at predefined thresholds: *p* < 0.05, *p* < 0.01, *p* < 0.001, and *p* < 0.0001. All data analyses were performed with at least three biological replicates and three technical replicates.

## 5. Conclusions

**Figure 13:**
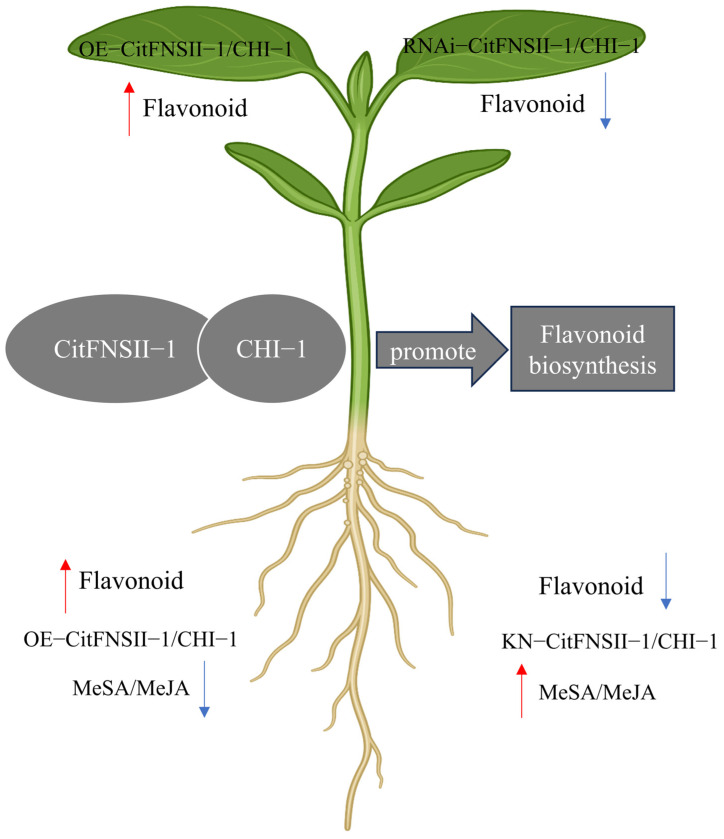
**A preliminary model illustrating the role of *CitFNSII-1* in flavonoid synthesis in citrus.** The CitFNSII-CHI complex positively regulates flavonoid biosynthesis. Red arrows indicate a significant increase; blue arrows indicate a significant decrease.

This study conducted systematic genome-wide identification and analysis of the FNSII gene family in citrus, identifying three FNSII family members. Evolutionary characterization revealed tandem duplication events within this family, suggesting tandem duplication as the primary mechanism driving family expansion. We analyzed the expression patterns of *CitFNSII-1* in citrus and functionally validated its predicted interacting protein CHI-1. Through transient expression techniques and *Agrobacterium rhizogenes*-mediated genetic transformation, we generated *CitFNSII-1*- and *CHI-1*-expressing transient expression leaves and transgenic hairy roots, followed by phytohormone quantification and flavonoid content analysis to investigate their roles in citrus flavonoid biosynthesis (Figure 13). The results revealed a theoretical foundation for the functional characterization of FNSII genes and provided a critical basis for molecular breeding applications in citrus improvement.

## Data Availability

The datasets generated during and/or analyzed during the current study are available from the corresponding author upon reasonable request.

## Supplementary Materials

The following supporting information can be downloaded at https://www.mdpi.com/article/10.3390/plants14131936/s1. Supplementary Table S1: The primers used in the study; Supplementary Figures file; Supplementary Figure S1: Analysis of the expression characteristics of *CitFNSII-1* gene in citrus; Supplementary Figure S2: Determination of *CitFNSII-1* transgenic and gene-edited hairy roots hormone content; Supplementary Figure S3: Analysis of the expression characteristics of *CHI-1* gene in citrus; Supplementary Figure S4: Determination of *CHI-1* transgenic and gene-edited hairy roots hormone content.
