# Supplementary material for: Genome-Wide Analysis of the FNSII Gene Family and the Role of CitFNSII-1 in Flavonoid Synthesis in Citrus
Source: Plants (Basel). 2025 Jun 24;14(13):1936. doi: 10.3390/plants14131936 (PMC12251898; doi:10.3390/plants14131936)
Supplement: Supplementary file 1 [file plants-14-01936-s001.zip › Supplementary figures.pdf]

Genome-wide analysis of the FNSII gene family and the role of CitFNSII-1 in flavonoid synthesis in citrus

Xinya Liu[1,4,5,†], Beibei Chen [2,†], Ling Luo[1,4,5,†], Qi Zhong[1,4,5], Chee How Teo[2,*], Shengjia Huang[3,*]

[1] Horticulture Research Institute, Sichuan Academy of Agricultural Sciences, Chengdu, People's Republic of China

[2] University Malaya Centre for Research in Biotechnology for Agriculture, Kuala Lumpur, MALAYSIA

[3] Institute of Economic Forest Research, Sichuan Academy of Forestry, Chengdu, People's Republic of China

[4] Key Laboratory of Horticultural Crops Biology and Germplasm Enhancement in Southwest, Ministry of Agriculture and Rural Affairs, Chengdu, People's Republic of China

[5] Key Laboratory for Germplasm Innovation & Utilization of Horticultural Crops of Sichuan Province, Chengdu, People's Republic of China

[†] These authors contributed equally to this work.

*Author to whom correspondence should be addressed.

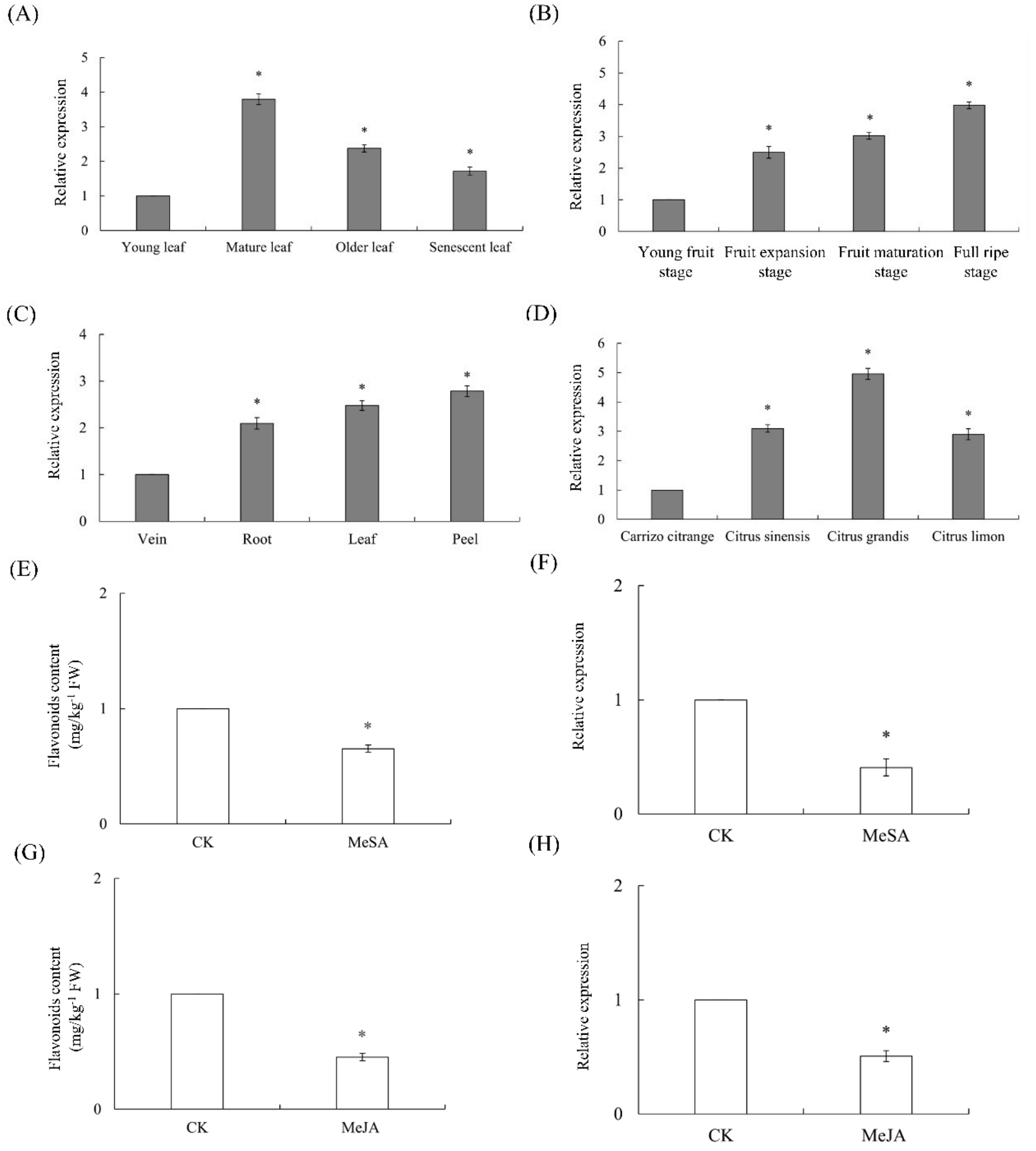

Supplementary Figure S1 | Analysis of the expression characteristics of *CitFNSII-1* gene in citrus. (A) Relative expression of *CitFNSII-1* in citrus leaves at different developmental stages. **(B)** Relative expression of *CitFNSII-1* in citrus fruits at different developmental stages. **(C)** Relative expression of *CitFNSII-1* in different tissues of citrus. **(D)** Relative expression of *CitFNSII-1* in different citrus cultivars. **(E)** Determination of flavonoid content in MeSA-treated citrus leaves. **(F)** Relative expression levels of *CitFNSIIs-1* in MeSA-treated citrus leaves. **(G)** Determination of flavonoid content in MeJA-treated citrus leaves. **(H)** Relative expression levels of *CitFNSIIs-1* in MeJA-treated citrus leaves. Values are expressed as means ± standard deviation of three independent tests. *on top of the bars indicates a significant difference ($p < 0.05$, Student's *t*-test).

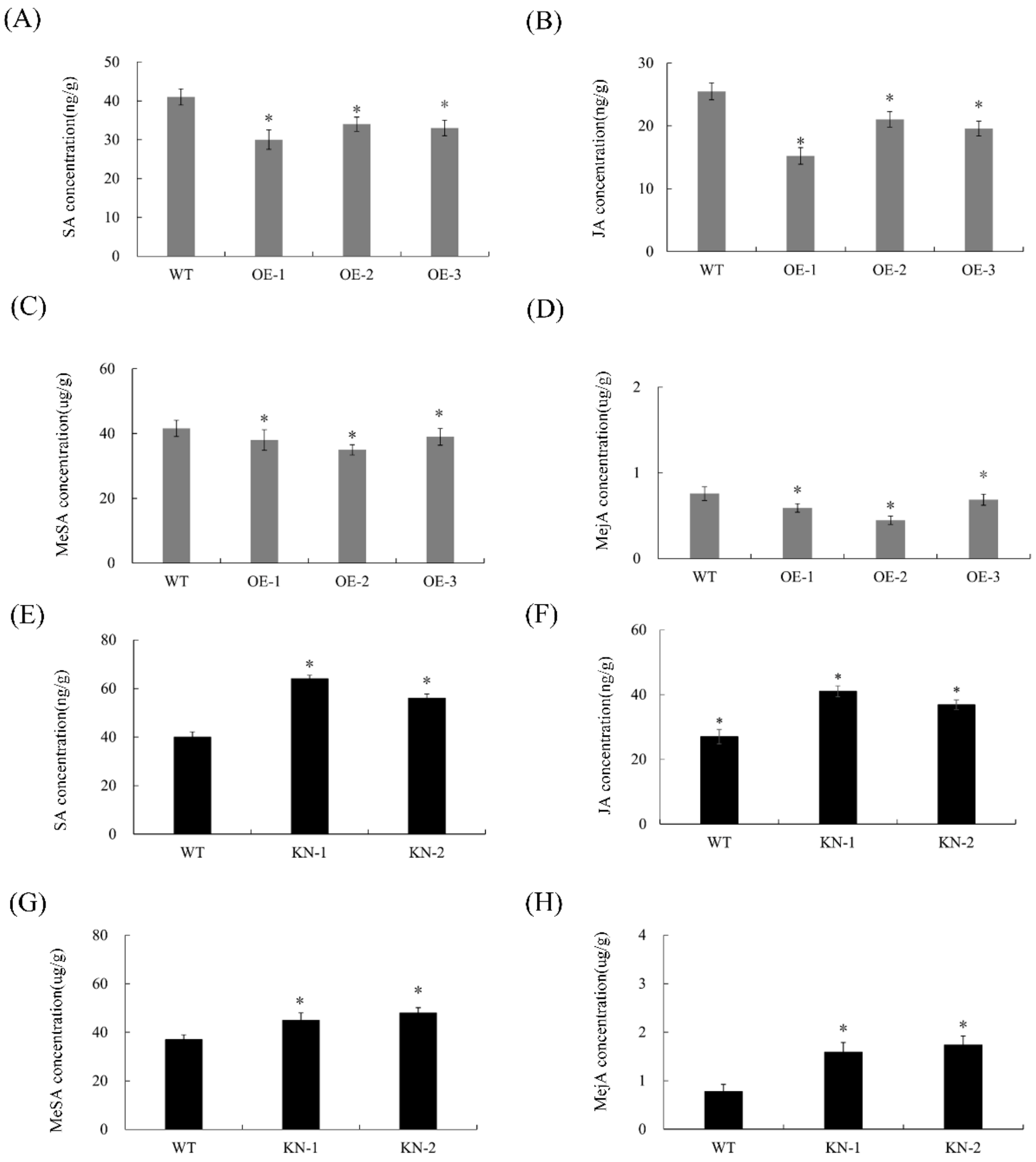

Supplementary Figure S2 | Determination of *CitFNSII-1* transgenic and gene-edited hairy roots hormone content. (A-D) Characteristics of SA, JA, MeSA and MeJA contents in *CitFNSII-1* transgenic hairy roots compared to WT control. **(E-H)** Characteristics of SA, JA, MeSA and MeJA contents in *CitFNSII-1* gene-edited hairy roots compared to WT control. Values are expressed as means ± standard deviation of three independent tests. *on top of the bars indicates a significant difference ($p < 0.05$, Student's *t*-test). WT, wild type; OE-#, transgenic hairy roots; KN-#, gene-edited hairy roots.

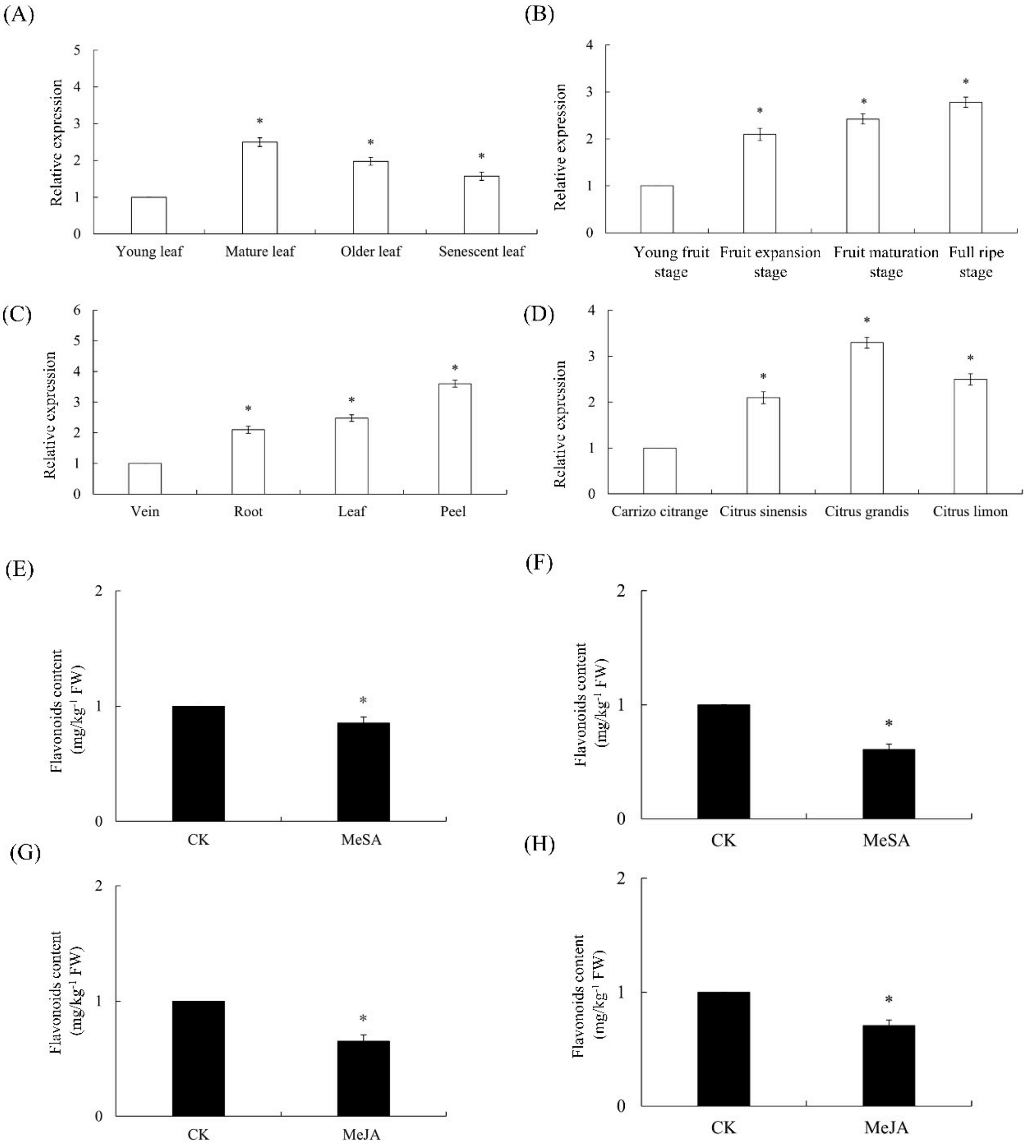

Supplementary Figure S3 | Analysis of the expression characteristics of *CHI-1* gene in citrus. (A) Developmental-stage-specific transcript abundance of *CHI-1*. **(B)** Expression dynamics of *CHI-1* during fruit maturation. **(C)** Spatial expression patterns of *CHI-1* across plant organs. **(D)** Phylogenetic comparison of *CHI-1* mRNA levels between different citrus varieties. **(E)** Determination of flavonoid content under MeSA treatment citrus leaves. **(F)** Relative expression levels of *CHI-1* under MeSA treatment citrus leaves. **(G)** Determination of flavonoid content under MeJA treatment citrus leaves. **(H)** Relative expression levels of *CHI-1* under MeJA treatment citrus leaves. Values are expressed as means ± standard deviation of three independent tests. *on top of the bars indicates a significant difference ($p < 0.05$, Student's *t*-test).

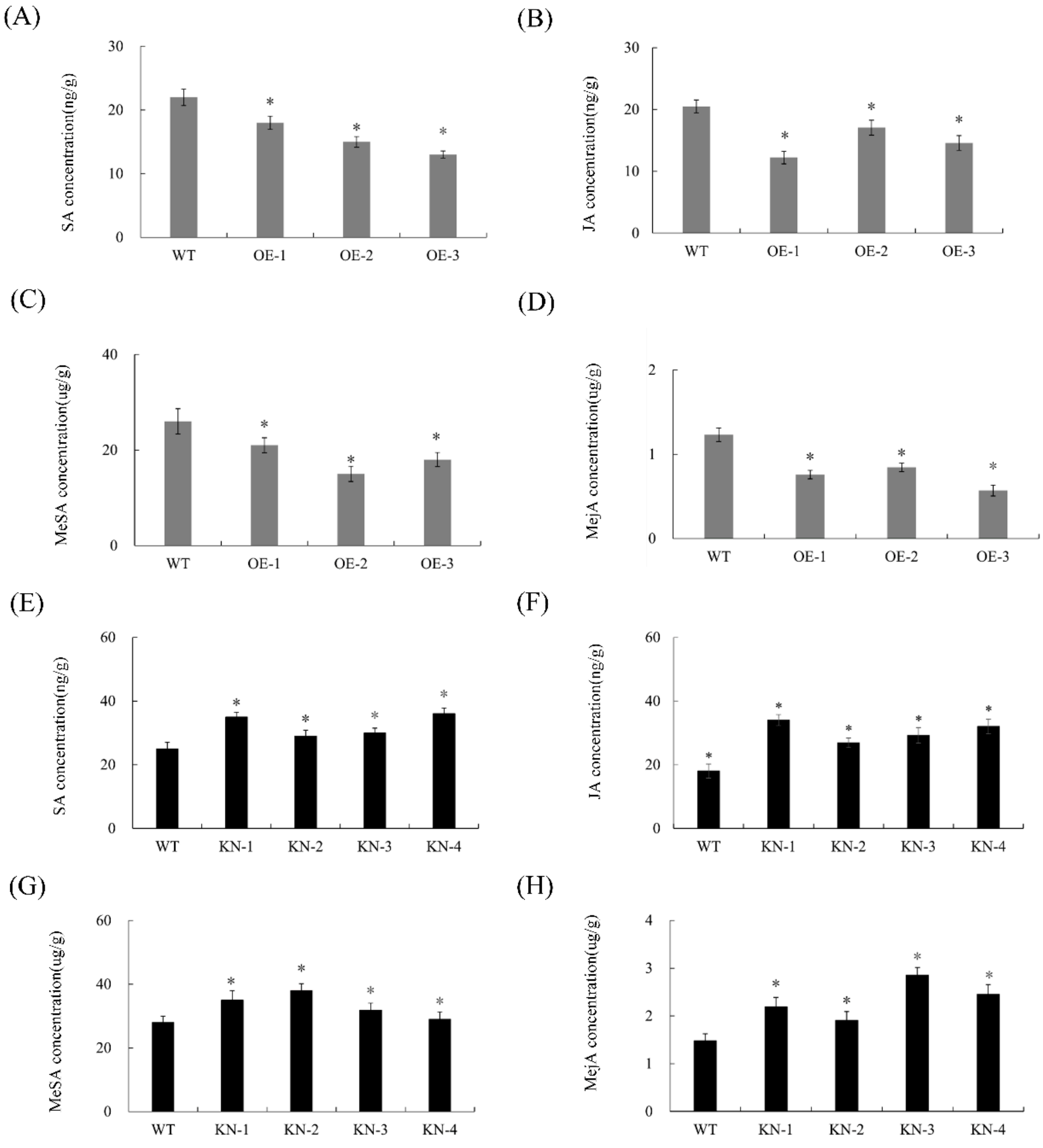

Supplementary Figure S4 | Determination of *CHI-1* transgenic and gene-edited hairy roots hormone content. (A-D) Characteristics of SA, JA, MeSA and MeJA contents in *CHI-1* transgenic hairy roots compared to WT control. **(E-H)** Characteristics of SA, JA, MeSA and MeJA contents in *CHI-1* gene-edited hairy roots compared to WT control. Values are expressed as means ± standard deviation of three independent tests. *on top of the bars indicates a significant difference ($p < 0.05$, Student's *t*-test). WT, wild type; OE-#, transgenic hairy roots; KN-#, gene-edited hairy roots.
